# Smac Mimetic SM-164 Potentiates APO2L/TRAIL- and Doxorubicin-Mediated Anticancer Activity in Human Hepatocellular Carcinoma Cells

**DOI:** 10.1371/journal.pone.0051461

**Published:** 2012-12-11

**Authors:** Shuijun Zhang, Gongquan Li, Yongfu Zhao, Guangzhi Liu, Yu Wang, Xiuxian Ma, Dexu Li, Yang Wu, Jianfeng Lu

**Affiliations:** 1 Department of Hepatobiliary and Pancreatic Surgery, the First Affiliated Hospital of Zhengzhou University, Zhengzhou, Henan, People’s Republic of China; 2 Key Laboratory of Hepatobiliary and Pancreatic Surgery & Digestive Organ Transplantation of Henan Province, Henan, People’s Republic of China; 3 Department of Obstetrics and Gynecology, Henan Provincial Hospital of Zhengzhou University, Zhengzhou, Henan, People’s Republic of China; 4 University of Michigan Comprehensive Cancer Center, University of Michigan, Ann Arbor, Michigan, United States of America; Bauer Research Foundation, United States of America

## Abstract

**Background:**

The members of inhibitor of apoptosis proteins (IAPs) family are key negative regulators of apoptosis. Overexpression of IAPs are found in hepatocellular carcinoma (HCC), and can contribute to chemotherapy resistance and recurrence of HCC. Small-molecule Second mitochondria-derived activator of caspases (Smac) mimetics have recently emerged as novel anticancer drugs through targeting IAPs. The specific aims of this study were to 1) examine the anticancer activity of Smac mimetics as a single agent and in combination with chemotherapy in HCC cells, and 2) investigate the mechanism of anticancer action of Smac mimetics.

**Methods:**

Four HCC cell lines, including SMMC-7721, BEL-7402, HepG2 and Hep3B, and 12 primary HCC cells were used in this study. Smac mimetic SM-164 was used to treat HCC cells. Cell viability, cell death induction and clonal formation assays were used to evaluate the anticancer activity. Western blotting analysis and a pancaspase inhibitor were used to investigate the mechanisms.

**Results:**

Although SM-164 induced complete cIAP-1 degradation, it displayed weak inhibitory effects on the viability of HCC cells. Nevertheless, SM-164 considerably potentiated Apo2 ligand or TNF-related apoptosis-inducing ligand (APO2L/TRAIL)- and Doxorubicin-mediated anticancer activity in HCC cells. Mechanistic studies demonstrated that SM-164 in combination with chemotherapeutic agents resulted in enhanced activation of caspases-9, -3 and cleavage of poly ADP-ribose polymerase (PARP), and also led to decreased AKT activation.

**Conclusions:**

Smac mimetics can enhance chemotherapeutic-mediated anticancer activity by enhancing apoptosis signaling and suppressing survival signaling in HCC cells. This study suggests Smac mimetics are potential therapeutic agents for HCC.

## Introduction

Human hepatocellular carcinoma (HCC) is a common aggressive malignancy and the 5th leading cause of cancer death worldwide [1]. Surgical resection, local treatment and liver transplantation may offer chances for a cure in only a small subset of HCC patients when diagnosis was made in the early stage. Nevertheless, a large majority of patients with advanced stage of HCC and compromised liver function depend on chemotherapy. Unfortunately, HCC is inherently resistant to chemotherapeutic agents, leading to a dismal prognosis for HCC patients. The major mechanisms that block the efficacy of chemotherapy in HCC include the defects of apoptosis program and the unwanted survival signaling, such as activation of AKT [2]–[5]. Therefore, it is imperative to explore novel drugs capable of overcoming chemotherapeutic resistance of HCC cells by removing these blockages.

Inhibitor of apoptosis proteins (IAPs) are a family of key apoptotic regulation proteins which are characterized by the presence of baculovirus IAP repeat domains (BIR) in their structure [6]–[8]. Accumulating evidence shows that IAPs are aberrantly overexpressed in HCC and many other types of cancers [9]–[15]. For instance, Shi et al. reported that X-linked IAP (XIAP), the best-characterized member of IAPs, was expressed at an elevated level in nearly 90% of clinical tumor samples from advanced HCC patients [9]. Moreover, since XIAP strongly inhibits caspases-9, and -3, two crucial apoptotic proteases with its BIR domains, XIAP confers resistance of HCC cells to Apo2 ligand or TNF-related apoptosis-inducing ligand (APO2L/TRAIL)- and chemotherapeutic-mediated apoptosis [9], [13]–[17]. Cellular IAP-1 (cIAP-1) and cellular IAP-2 (cIAP-2) are another two potent IAP family members [6]–[8]. Although cIAP-1 and cIAP-2 exhibit weak potency in inhibiting caspases-9 and -3, it was revealed recently that these two IAPs inhibit apoptosis by preventing the death-receptors complex formation and caspase-8 activation [16]–[18]. Besides these antiapoptotic functions, IAPs were found involved in maintaining cell survival *in vitro* and metastatic dissemination *in vivo* in breast cancer MDA-MB-231 and prostate cancer PC3 tumor models [19]–[20]. Therefore, IAP proteins represent promising targets for human cancer treatment.

IAPs can be bound and antagonized by Second mitochondria-derived activator of caspases (Smac), a 25 KD protein released from mitochondria during apoptosis. The antagonism of IAPs by Smac subsequently relieves the inhibition of caspases by IAPs and leads to apoptosis [21]–[23]. Accordingly, molecules that mimic the binding interactions between IAPs and Smac, referred to as Smac mimetics, are being designed as a novel class of anticancer drugs through targeting IAP proteins. Up to now, a number of Smac mimetics with strong anticancer activities have been reported [16], [24]–[26]. SM-164 is a potent cell-permeable Smac mimetic. Biochemical studies showed that SM-164 binds to a XIAP protein with a Ki value of 0.56 nM, and binds to cIAP-1 and cIAP-2 proteins with Ki values of 0.31 and 1.1 nM, respectively [26]–[27]. SM-164 has been widely used in anticancer studies [17], [26]–[27]. It has been shown that SM-164 elicits strong anticancer activity in multiple types of human cancers, including breast cancer, colon cancer, prostate cancers and ovarian cancer [17], [27]. We therefore investigated the anticancer action of Smac mimetics in human HCC cells using SM-164. We found that SM-164 not only sensitizes HCC cells to APO2L/TRAIL, but also greatly potentiates the cytotoxic effect of Doxorubicin, a standard chemotherapeutic drug on HCC cells. Our results suggest Smac mimetics are potential therapeutic agents for human HCC.

**Figure 1:**
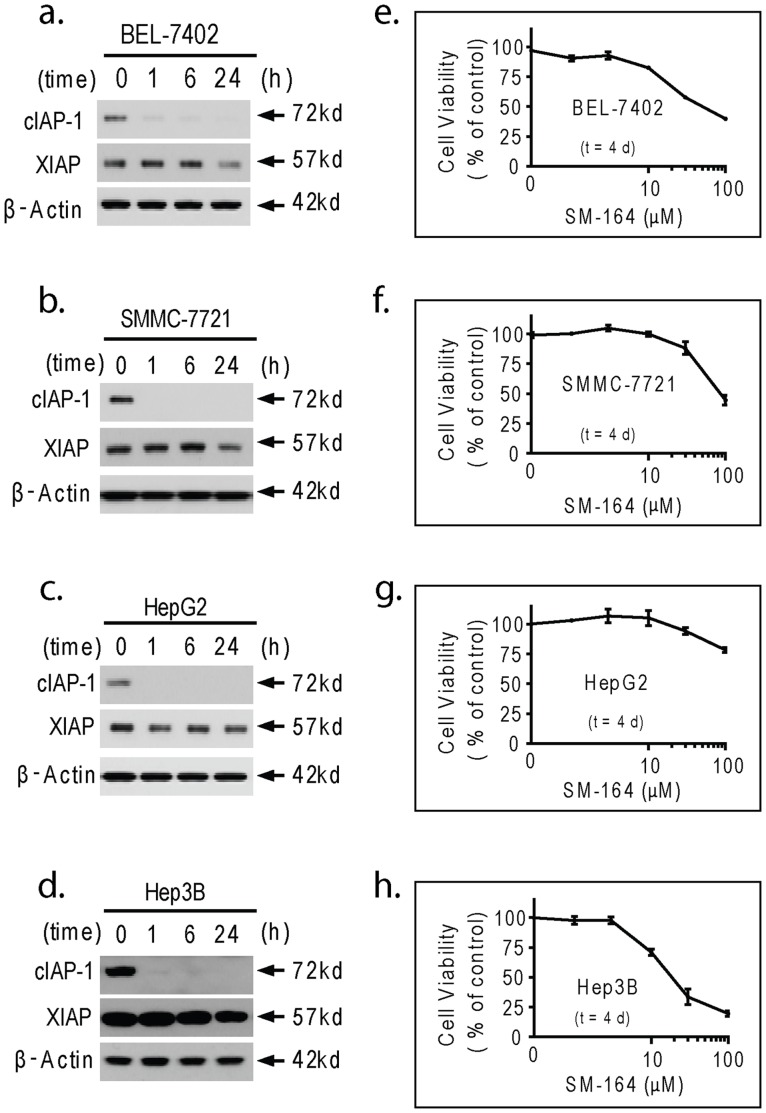
SM-164 induces rapid cIAP-1 degradation and displays modest single-agent effect in HCC cells. (a–d). BEL-7402, SMMC-7721, HepG2 and Hep3B cell lines were treated with SM-164 at 0.1 µM for 1, 6 and 24 h. Whole cell lysates were examined for the expressions of cIAP-1 and XIAP by western blotting analysis and specific antibodies. β-Actin was used as a loading control. (e-h). BEL-7402, SMMC-7721, HepG2 and Hep3B cell lines were treated with SM-164 for 4 d, cell viability inhibition was determined using an MTT assay.

**Figure 2:**
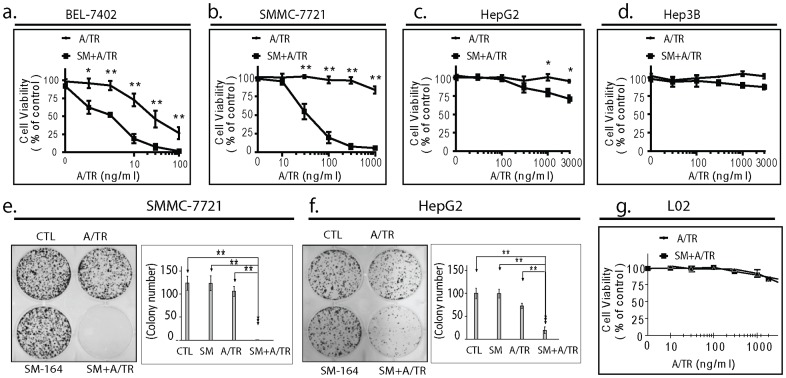
SM-164 enhances APO2L/TRAIL-mediated anticancer activity in HCC cells. (a–d). BEL-7402, SMMC-7721, HepG2 and Hep3B cell lines were treated with APO2L/TRAIL (A/TR) alone, or in combination with SM-164 at 0.1 µM (SM+ A/TR) for 4 d, cell viability inhibition was determined using an MTT assay. (e-f). SMMC-7721 and HepG2 cells were seeded into six-well plates at 600 cells per well in triplicates, and treated with SM-164 alone, A/TR alone or their combination (SM+ A/TR) for 2 weeks, followed by 0.05% methylene blue staining and colony counting. (left panels), representative results show photographs of stained 6-well plates for SMMC-7721 and HepG2, respectively. (right panels), data show means ± S.D. Normal human liver cell line L02 was treated as indicated for 4 d, cell viability inhibition was determined using an MTT assay. *, p<0.05, * *, p<0.01.

**Figure 3:**
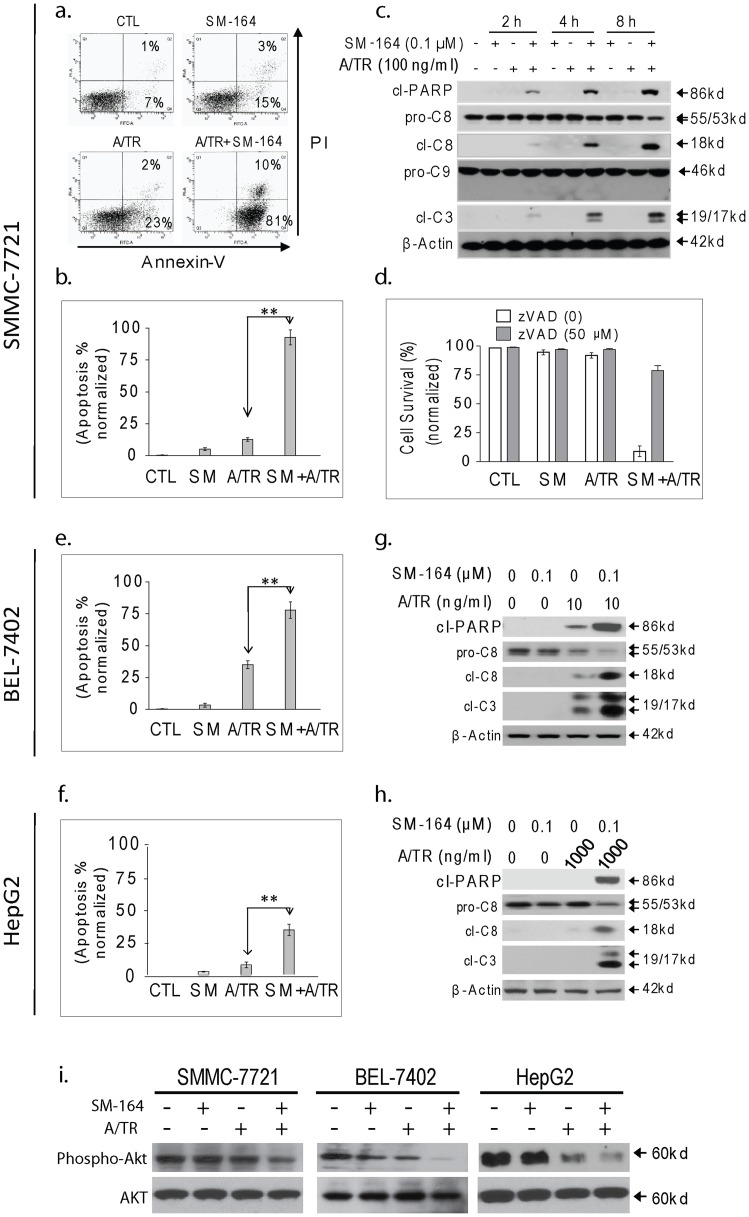
SM-164 promotes APO2L/TRAIL-induced apoptosis and inhibits AKT activation in human HCC cell lines. (a–b). SMMC-7721 cell line was treated with SM-164 (SM) at 0.1 µM alone, APO2L/TRAIL (A/TR) at 100 ng/ml alone or both for 16 h, cells were stained with Annexin V/PI staining and examined by flow cytometry assay. (a). Representative dot plot of apoptosis assay. (b). data of Annexin V-positive cells show means ± S.D. of from three experiments. **, p<0.01. (c). SMMC-7721 cell line was treated as indicated, whole cell lysates were analyzed with western blotting and specific antibodies as indicated. β-Actin was used as a loading control. (d). SMMC-7721 cell line was pretreated with pan-caspases inhibitor, zVAD (50 µM) for 1 h and followed by treatment with SM at 0.1 µM alone, A/TR at 100 ng/ml alone or both for 24 h, cell death induction was examined with trypan blue exclusion assay. (e). BEL-7402 cell line treated with SM at 0.1 µM alone, A/TR at 10 ng/ml or both for 16 h was examined with Annexin V/PI staining and flow cytometry assay. (f). HepG2 cell line treated with SM at 0.1 µM alone, A/TR at 1000 ng/ml or both for 16 h was examined with Annexin V/PI staining and flow cytometry assay. (g-h). BEL-7402 and HepG2 cell lines were treated as indicated for 12 h. Whole cell lysates were analyzed with western blotting and specific antibodies. (i). Cell lines were treated as indicated for 12 h. Whole cell lysates were analyzed with western blotting and specific antibodies. β-Actin was used as a loading control.

**Figure 4:**
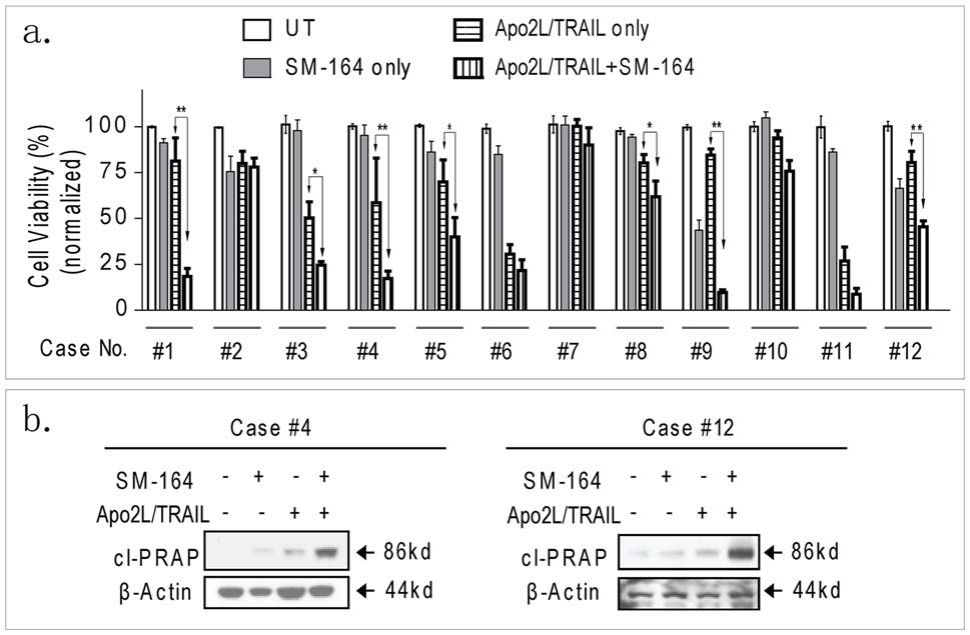
SM-164 potentiates cell viability inhibition by APO2L/TRAIL in primary HCC cells. (a). Primary HCC cells freshly isolated from 12 patients’ samples were treated with SM-164 at 0.1 µM alone, APO2L/TRAIL at 100 ng/ml alone or both for 24 h, cell viability was determined with trypan blue exclusion assays. *, p<0.05, * *, p<0.01. (b). Primary HCC cells from Case No.4 and No.12 patients’ samples were treated with SM-164 at 0.1 µM alone, APO2L/TRAIL at 100 ng/ml alone or both for 24 h. Whole cell lysates were probed with anti-cleaved PARP (cl-PARP). β-Actin was used as a loading control.

**Table 1:** Clinical data of HCC patients.

Case No.	Sex	Age (years)	Hepatitis Virus	Tumor Volume (mm)	Pathology Diagnosis	Stage	Liver Function	Significance
1	m	50	HCV (+)	151X41	HCC	III b	II	++
2	m	32	HBsAg(+)	58X63	HCC	III a	I	–
3	m	42	HBsAg(+)	43X41	HCC	II	I	+
4	f	42	HBsAg(+)	35X27	HCC	II	II	++
5	m	72	HBsAg(+)	60X65	HCC	III a	I	+
6	m	60	HBsAg(+)	103X91	HCC	IV	I	–
7	f	52	HBsAg(+)	35X32	HCC	III a	II	–
8	m	55	HBsAg(+)	27X31	HCC	I	II	+
9	m	35	HBsAg(+)	51X44	HCC	III b	I	++
10	m	48	HCV (+)	72X52	HCC	III b	II	–
11	f	52	HBsAg(+)	33X29	HCC	II	I	–
12	m	47	HBsAg(+)	62X38	HCC	III b	II	++

**Figure 5:**
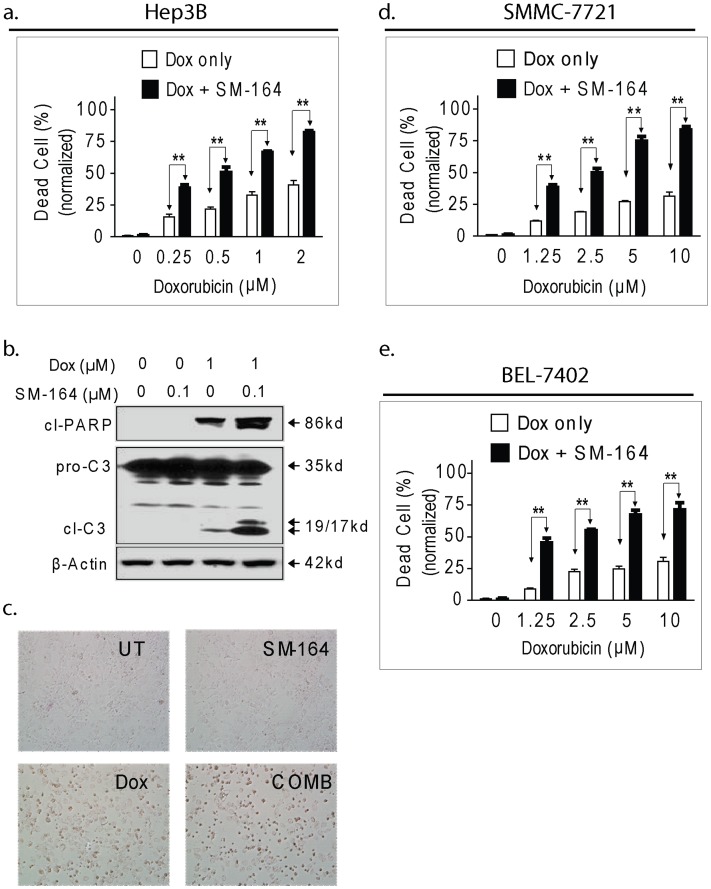
SM-164 potentiates the cytotoxic effect of Doxorubicin in HCC cells. (a). Hep3B cell line was treated with Doxorubicin (Dox) alone, or in combination with SM-164 at 0.1 µM for 48 h, cell death induction was determined in a trypan blue excluson assay; (b). Hep3B cell line was treated as indicated, whole cell lysates were analyzed with western blotting and specific antibodies. β-Actin was used as a loading control. (c). Hep3B cell line was treated with Dox at 1 µM alone, or SM-164 at 0.1 µM alone for 48 h, representative micrographs were shown. (d-e). SMMC-7721 and BEL-7402 cell lines were treated as indicated, cell death induction was determined in trypan blue exclusion assays;

**Figure 6:**
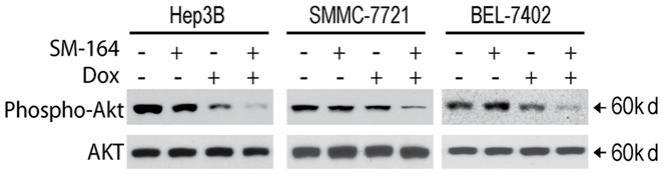
SM-164 inhibits AKT activation in HCC cells. Cells were treated with SM-164 at 0.1 µM alone, Dox alone (1 µM for Hep3B, 10 µM for SMMC-7721 and BEL-7402) for 24 h. Whole cell lysates were probed with anti-phospho-AKT and then re-probed with anti-AKT (unphosphorylated) which served as a loading control.

## Materials and Methods

### Reagents and Antibodies

SM-164 was designed and synthesized at the University of Michigan [26]. APO2L/TRAIL was purchased from PeproTech Inc. (Shanghai, China). Pancaspase inhibitor zVAD-fmk was purchased from Sigma (Shanghai, China). The following primary antibodies were used in the study: anti-XIAP, anti-cleaved poly ADP-ribose polymerase (PARP), anti-procaspase-8, anti-cleaved caspase-8, anti-cleaved caspase-3, anti-phospho-AKT and anti-AKT from Cell Signaling Technology Shanghai Biological Reagents Company (Shanghai, China). Anti-cIAP-1 was from R&D Systems (Shanghai, China).

### Cell Lines and Cell Culture

Human HCC SMMC-7721, BEL-7402 cell lines and human normal liver cell line L02 were purchased from the Cell Bank of Type Culture Collection of Chinese Academy of Sciences (Shanghai, China). HepG2 and Hep3B were purchased from the American Type Culture Collection (Manassas, VA). All 5 cell lines were maintained in RPMI 1640 (Invitrogen, Shanghai, China) supplemented with 10% fetal bovine serum (Invitrogen) and 1% penicillin-streptomycin at 37°C in a humidified atmosphere containing 5% CO2 in air. Using a Mycoplasma Detection Kit (Roche, Shanghai, China), we have tested and confirmed that there is no mycoplasma contamination in all cell lines used in this study.

### Cell Viability Inhibition, Cell Death Induction and Apoptosis

Cell viability inhibition was evaluated by a colorimetric assay based on 3-(4,5-dimethylthiazol-2-yl)-2,5-diphenyl tetrazolium bromide (MTT). Briefly, cells were seeded in 96-well plates at 20–30% confluence. After culture overnight, cells were treated with various concentrations of APO2L/TRAIL and/or SM-164 for 4 d. At the end of treatment, MTT dye was added to each well and plates were incubated at 37°C for 4 h. The supernatants were decanted. Insoluble formazan complexes were solubilized with Dimethyl sulfoxide (DMSO) and the absorbance was measured at 540 nm using a Benchmark Plus microplate spectrophotometer (Bio-Rad, Hercules, CA). Cell viability inhibition was evaluated as the ratio of the absorbance of the samples to that of the control. Each experimental condition was done in triplicate and performed at least twice. Besides, we found that SM-164, APO2L/TRAIL or both have no influence on MTT assay reagents in the absence of cells. Cell death induction was quantitated by microscopic examination in a trypan blue excluson assay. Apoptosis analysis was done using an Annexin V/propidium iodide (PI) apoptosis detection kit (Roche, Shanghai, China) by flow cytometry according to the manufacturer's instructions. Cells positively stained with Annexin V were counted as apoptotic cells.

### Ethics Statement

The study was approved by the Ethics Committee of the First Affiliated Hospital of Zhengzhou University (no.2010-02-008). Informed, written consent was obtained from all participants or their legal guardians.

### Primary HCC Cells Isolation, Culture and Treatment

Fresh HCC tissues were obtained from the surgical specimens at the Department of Hepatobiliary and Pancreatic Surgery of the First Affiliated Hospital of Zhengzhou University. The liver cancer tissue was scraped directly for culture, or minced into small pieces (0.5–1.5 mm^3^ in size), and digestion with collagenase (type IV, 0.1%) (Amresco, JingKeHongDa Biotechnology Co., Beijing, China), hyaluronidase (type V, 0.01%) (Amresco), and DNase (type I, 0.002%) (Amresco) for 1–3 h. The single cell suspension was isolated by Percoll gradient centrifugation (GE Healthcare, Beijing, China) and cultured in RPMI 1640 with 10–15% fetal bovine serum, supplemented with recombinant human epidermal growth factor (EGF, 30 ng/ml) (R&D system, Shanghai, China).

### Western Blotting Analysis

Cells were lysed using radioimmunoprecipitation assay lysis buffer (PBS containing 1% NP40, 0.5% Na-deoxycholate, and 0.1% SDS) supplemented with 1 µmol/L phenylmethylsulfonyl fluoride and 1 protease inhibitor cocktail tablet *per* 10 mL on ice for 20 min, and lysates protein concentration were determined using the Bio-Rad protein assay kit according to the manufacturer's instructions. Proteins were electrophoresed onto 4–20% SDS-PAGE gels (Invitrogen, Carlsbad, CA) and transferred onto polyvinylidene difluoride membranes. Following blocking in 5% milk, membranes were incubated with a specific primary antibody, washed, and incubated with horseradish peroxidase–linked secondary antibody (GE Healthcare, Beijing, China). Signals were visualized with chemiluminescent horseradish peroxidase antibody detection reagent (Denville Scientific, Guangzhou, China).

### Colony Formation Assay

Cells were split and seeded into six-well plates at 600 cells per well in triplicate, followed by incubation for 14 d. The colonies were stained with 0.05% methylene blue, and counted.

### Microscopy

Exponentially growing Hep3B cells were treated with SM-164 alone, Doxorubicin alone or their combination for 48 h. Cells were examined for morphological changes under light microscope.

### Statistical Analysis

Statistical analyses were performed by one-way ANOVA using SPSS (version 13.0, SPSS Inc, Illinois, USA). P<0.05 was considered statistically significant, P<0.01 was considered very statistically significant.

## Results

### 1. SM-164 as a Single Agent Displays Modest Effect on HCC Cells

We first investigated the effect of Smac mimetic SM-164 on the levels of both cIAP-1 and XIAP in BEL-7402, SMMC-7721 HepG2 and Hep3B HCC cell lines with western blotting analysis. We found that 0.1 µM of SM-164 induced complete cIAP-1 degradation within 1 h, but had minimal effect on the level of XIAP even after 24 h in these HCC cell lines (Fig. 1a–d). We next tested the effect of SM-164 on cell viability in HCC cell lines using an MTT cell assay. We noted that treatment with SM-164 for 4 d had only minimal to modest inhibition effect on these 4 HCC cell lines (Fig. 1e–h). Hep3B was most sensitive cell line, with an IC50 value of 21 µM. HepG2 was the most resistant, SM-164 even at 100 µM could not achieve 50% cell viability inhibition. Moreover, the IC50 values of SM-164 in SMMC-7721 and BEL-7402 were 63 and 45 µM, respectively. These results suggested that in line with cancer cells from other tumor types, HCC cell lines are relative resistant to Smac mimetics [13]–[15], [27].

### 2. SM-164 Potentiates HCC Cells to APO2L/TRAIL-mediated Cell Viability Inhibition

Next, we examined whether SM-164 could potentiate APO2L/TRAIL-mediated cell viability inhibition in HCC cell lines. We first screened APO2L/TRAIL-sensitivity in BEL-7402, SMMC-7721, HepG2 and Hep3B HCC cell lines using an MTT assay. We found that BEL-7402 cell line was sensitive to APO2L/TRAIL. Treatment with 10, 30 and 100 ng/ml of APO2L/TRAIL for 4 d inhibited cell viability by 27%, 54% and 74%, respectively (Fig. 2a). SMMC-7721, HepG2 and Hep3B cell lines were resistant to APO2L/TRAIL. Treatment with 1,000 ng/ml of APO2L/TRAIL for 4 d induced a limited cell viability inhibition (<10%) in all these 3 cell lines (Fig. 2b–c). We then examined the combinational effect of APO2L/TRAIL with SM-164 in these HCC cell lines. In BEL-7402 cell line, SM-164 at 0.1 µM, a nontoxic concentration, significantly enhanced APO2L/TRAIL-mediated cell viability inhibition at all 5 concentrations of APO2L/TRAIL employed (p<0.05). For instance, APO2L/TRAIL at 10 ng/ml inhibited cell viability by 27%. By contrast, APO2L/TRAIL in combination with SM-164 inhibited cell viability by 83%, showing SM-164 potentiated APO2L/TRAIL activity in sensitive HCC cells (Fig. 2a). We also noted that SM-164 considerably enhanced APO2L/TRAIL-mediated cell viability inhibition in resistant SMMC-7721 and HepG2 cell lines (Fig. 2b–c). Particularly, in SMMC-7721 cell line, APO2L/TRAIL alone at 1,000 ng/ml had modest effect, while APO2L/TRAIL in combination with SM-164 achieved about 80% and 100% cell viability inhibition when APO2L/TRAIL concentrations were 100 and 300 ng/ml, respectively (Fig. 2b). Moreover, we observed that SM-164 modestly enhanced APO2L/TRAIL-activity in Hep3B cell line, despite the effect lacked significance (p>0.05) (Fig. 2d). Thus, SM-164 was able to convert APO2L/TRAIL-resistant HCC cells to become sensitive.

### 3. SM-164 Dramatically Potentiates APO2L/TRAIL-mediated Colony Formation in HCC Cell Lines

Colony assays were performed to determine whether the combination of SM-164 and APO2L/TRAIL had long-term anticancer activity in HCC cells. Greater inhibition of colony formation was observed at 2 weeks when APO2L/TRAIL-resistant SMMC-7721 and HepG2 cell lines were exposed to the combination therapy versus either modality alone (Fig. 2e–f). In SMMC-7721 cell line, whereas neither single agent therapeutic modality showed clear efficacy at the concentrations used, the combination achieved complete inhibition of colony formation (P<0.001) (Fig. 3e). Moreover, in HepG2 cell line, despite the combination showed a modest effect on inhibition of cell viability and induction of apoptosis (Fig. 2–3), the same combination resulted in dramatic inhibition of colony formation as compared to treatment with either single agent (P<0.001) (Fig. 3f). Thus, our results demonstrated that SM-164 in combination with TRAIL displayed a long-term effect on inhibition of HCC cell survival and proliferation.

### 4. SM-164 does not Increase the Toxicity of APO2L/TRAIL to Human Normal Liver Cells

We next tested whether SM-164 enhances the toxicity of APO2L/TRAIL to human normal liver cells using L02 cell line. We noted that APO2L/TRAIL is slightly toxic to L02 cells. For instance, cell viability WST assays showed that treatment with APO2L/TRAIL alone at 300 ng/ml and 3,000 ng/ml for 4 days inhibited cell viability by 7% and 23%, respectively. We further noted that SM-164 at 0.1 µM did not enhance APO2L/TRAIL-mediated cell viability inhibition (Fig. 2g). These results suggest Smac mimetics do not increase the toxicity of APO2L/TRAIL to human normal liver cells.

Collectively, these results demonstrated Smac mimetic SM-164 significantly potentiates APO2L/TRAIL-mediated anticancer activity in HCC cells with minimal toxicity on human normal liver cells.

### 5. SM-164 Potentiates APO2L/TRAIL-triggered Apoptosis in Human HCC Cell Lines

It has been shown that the synergism of Smac mimetics with APO2L/TRAIL in cancer cells of other tumor types was primarily mediated by apoptotic pathway [16]–[17], [28]–[30]. To investigate whether there was a similar mechanism in HCC cells, we next investigated apoptosis induction by APO2L/TRAIL in combination with SM-164 in HCC cell lines. In an Annexin-V staining and flow cytometry assay for SMMC-7721 cell line, we found that as compared to untreated control, treatment with SM-164 alone or APO2L/TRAIL alone for 24 h increased apoptotic cells by 7% and 14%, respectively. In strikingly contrast, their combination increased apoptotic cells by 77% (Fig. 3a–b). To further confirm the phenomena observed above, western blotting analysis were preformed to assess apoptosis markers. Consistently, we noted that SM-164 markedly enhanced APO2L/TRAIL-triggered activation of initiator caspase-8, effector caspase-3 and accumulation of cleaved PARP (Fig. 3c). Moreover, cell death induction by the combination was dramatically attenuated by pretreatment with pancaspase inhibitor z-VAD-fmk (Fig. 3d). In addition, dramatic enhancement of APO2L/TRAIL-mediated apoptosis by SM-164 was also observed in BEL-7402 and HepG2 cell lines (Fig. 3e–h). These results suggest that SM-164 enhances APO2L/TRAIL-mediated anticancer activity *via* caspase-dependent apoptosis pathway in human HCC cells.

### 6. SM-164 in Combination with APO2L/TRAIL Inhibits AKT Activation in HCC Cells

We next examined the effect of the combination on the activation of pro-survival protein AKT in SMMC-7721, BEL-7402 and HepG2 cell lines. Cells were treated with SM-164 alone, APO2L/TRAIL alone, or both. Whole cell lysates were probed with anti-phospho-AKT and then re-probed with anti-AKT (unphosphorylated) which served as a loading control. As shown in Fig. 3i, constitutive activation of AKT was observed in all three HCC cell lines. Moreover, we found that there was no noticeable modification of phosphorylation of AKT following treatment by SM-164. Treatment with APO2L/TRAIL alone at the indicated concentrations modestly inhibited AKT activation. However, treatment by APO2L/TRAIL in combination with SM-164 markedly reduced the level of phosphorylation of AKT, indicating the combination suppresses cell survival signaling.

### 7. SM-164 Potentiates APO2L/TRAIL-mediated Cell Death Induction in Primary HCC Cells

To more accurately assess the clinical relevance of the combination, we examined the response of primary HCC cells to APO2L/TRAIL, SM-164 and their combination. Primary cultured HCC cells from 12 patients were tested. Clinical data of HCC patients are summarized in Table 1. Among the 12 patients, 9 are male and 3 are female, aged from 42 to 72 years. All 12 patients had a solitary HCC tumor. Tumor diameters range from 35 mm to 151 mm. No patients received chemotherapy or transarterial chemoembolization before operation. After single cell suspension isolation, primary HCC cells were cultured overnight for recovery from stress caused by cells isolation, then cells with high viability (90%–95%) were treated for 24 h with SM-164 (0.1 µM) alone, APO2L/TRAIL (100 ng/ml) alone, and their combination, and analyzed for viability. Consistent with findings from HCC cell lines, most primary HCC cells were insensitive to SM-164. SM-164 induced obvious cell death only in 2 cells (No.9 and No.12). Nevertheless, SM-164 enhanced APO2L/TRAIL-mediated cell death induction in HCC cells from 11 out of 12 patients, including APO2L/TRAIL-sensitive cells from 5 patients and APO2L/TRAIL-resistant cells from 6 patients. Importantly, enhancement in cells from 7 patients showed statistically significance (p<0.05). Moreover, the cell death induction of primary HCC cells by the combination was mediated *via* apoptosis pathway as demonstrated by PARP cleavage in the western blotting analysis (Fig. 4b). However, it appeared that APO2L/TRAIL-sensitization by SM-164 was not correlated with disease stages.

Taken together, the results from HCC cell lines and primary HCC cells demonstrated that Smac mimetics substantially potentiate APO2L/TRAIL-mediated anticancer activity in human HCC cells.

### 8. SM-164 Potentiates the Cytotoxic Effect of Doxorubicin in HCC Cells

It was reported recently that Smac mimetics improve the efficacy of standard chemotherapies in human lung cancer, pancreatic cancer and leukemia cells [31]–[34]. We thus next examined whether SM-164 enhanced anti-HCC activity of Doxorubicin, the most commonly used chemotherapy for treatment of advanced HCC using HCC cell lines. We observed that HCC cell lines displayed heterogeneous sensitivity to cell death induction by Doxorubicin (Fig. 5). Hep3B cell line was most sensitive, with 41% of cell death induction when treated with 1 µM of Doxorubicin for 48 h. SMMC-7721 and BEL-7402 cell lines were relative resistant, with only 32%, 31% of cell death induction at 10 µM of Doxorubicin, respectively. Nonetheless, SM-164 at 0.1 µM significantly enhanced Doxorubicin-mediated cell death induction in all these three HCC cell lines. For example, in Hep3B cell line, 0.25 µM of Doxorubicin in combination with SM-164 reached same effect as with the treatment of 2 µM of Doxorubicin alone (Fig. 5a). Furthermore, more shrunk cells and floating cells were observed under microscope in cells treated with the combination than in cells treated with single agents (Fig. 5b). Western blotting analysis showed that SM-164 markedly enhanced Doxorubicin-mediated activation of caspase-3 and cleaved PARP accumulation in this HCC cell line (Fig. 5c). Similar combination effect was also found in SMMC-7721 and BEL-7402 cell lines (Fig. 5d–e). Doxorubicin at 1.25 µM in combination with SM-164 achieved similar effect of cell death induction as with the treatment of 10 µM of Doxorubicin alone in these two cell lines. Thus, these results demonstrate that SM-164 potentiates the cytotoxic effect of Doxorubicin in these three HCC cell lines.

### 9. SM-164 in Combination with Doxorubicin Inhibits AKT Activation in HCC Cells

Next we investigated the effect of SM-164, Doxorubicin and their combination on pro-survival protein AKT. HCC cells were treated with SM-164, Doxorubicin alone or both for 24 h, AKT level in cell lysates were examined with western blotting analysis. We observed that either SM-164 or Doxorubicin as a single agent had little or modest effect on AKT phosphorylation. However, we noted that level of phosphor-AKT was markedly reduced following combination treatment in all these 3 cell lines (Fig. 6). In particular, in Hep3B and BEL-7402 cell lines, the phosphorylated form of Akt was barely detectable following the combination treatment, implying that combination treatment with SM-164 and Doxorubicin strongly inhibits pro-survival signaling in HCC cells.

## Discussion

IAPs play a critical role in suppressing apoptosis, attributed to its capacity to control the function of key components of apoptosis machineries, such as caspases [6]–[8], [9]–[15]. It has been shown that IAP family proteins are overexpressed in HCC, and confer resistance of HCC cells to chemotherapeutic agents. Suppression of XIAP with small RNA interference technique augmented the cytotoxic effect of APO2L/TRAIL and standard chemotherapies on HCC cells, suggesting that suppression of IAPs is a novel strategy for human HCC treatment [9], [12].

In this study, we investigated the anti-HCC activity of SM-164, a drug-like small molecule Smac mimetic with capacity to concurrently target multiple IAP members. Our data demonstrated that SM-164 is able to greatly potentiate APO2L/TRAIL-mediated anticancer activity in HCC cells. In agreement with findings in cancer cells of other tumor types [16]–[17], [29]–[34], we observed that SM-164 not only improved APO2L/TRAIL-activity in sensitive BEL-7402 cell line, but also converted APO2L/TRAIL-resistant SMMC-7721 and HepG2 cell lines to become sensitive. Notably, in APO2L/TRAIL-resistant SMMC-7721 line, the combination of SM-164 and APO2L/TRAIL could cause complete cell viability inhibition. Moreover, the combination also displayed strong effects on suppressing clonogenic survival in two APO2L/TRAIL-resistant HCC cell lines, showing a long-term anticancer activity. More importantly, we found that SM-164 significantly enhanced APO2L/TRAIL-mediated cell death induction in 7 out of 12 of fresh isolated HCC cells from tumor samples, highlighting the clinical relevance of using Smac mimetics cooperated with APO2L/TRAIL in human HCC therapy.

We elucidated that the underlying mechanism of SM-164-mediated APO2L/TRAIL-sensitization was mainly through the enhancement of caspases activation and apoptosis induction. This conclusion was strongly supported by the functional rescue experiments in which inactivation of caspases using a pharmaceutical (z-VAD) attenuates the sensitization. In particular, we observed activation of initiator caspase-8 was markedly enhanced in the combination; by contrast, activation of initiator caspase-9 was only minimal increased. Thus, these evidences indicated that, in line with our finding in breast cancer cells [17], the combination effect on HCC cells was largely mediated by the extrinsic apoptosis pathway.

However, as shown previously in the studies with breast cancer, ovarian cancer, prostate cancer and other cancer cell lines, the combination of APO2L/TRAIL with Smac mimetics was effective only in some, but not all HCC cells [17], [28]–[30]. This may reflect the complex mechanisms that regulate APO2L/TRAIL-mediated apoptosis signaling. It is known that several antiapoptotic proteins, such as cellular FLICE-like inhibitory protein (c-FLIP) and antiapoptosis Bcl-2 family protein Mcl-1 play a critical role in regulating APO2L/TRAIL-sensitivity [29], [35]. We deduced that c-FLIP, Mcl-1 and other antiapoptotic factors may also play a role in regulation of sensitivity of HCC cells to the combination, and we will investigate this in our future studies.

Doxorubicin is the most commonly used standard chemotherapy for HCC [36]–[37]. Doxorubicin causes DNA damage and kills cancer cells at least in part by inducing apoptosis. However, HCC may partially or completely resist to the treatment of Doxorubicin as a single agent due to the defects of apoptosis program in HCC cells [38]. In this study, we demonstrated that SM-164 potentiated the cytotoxic effect of Doxorubicin in HCC cells by overcoming apoptosis resistance. There were several lines of evidence that support this conclusion. (1) Cell viability and morphological examination assays showed that as compared with single agents, combination treatment caused more cancer cell losing viability; (2) Western blotting analysis showed that SM-164 markedly augmented Doxorubicin-mediated PARP cleavage and caspase-3 activation, two key biomarkers for apoptosis induction. These findings indicated that using Smac mimetics as a sensitizer in Doxorubicin-based combination regimens might be a novel strategy to enhance anticancer efficacy in human HCC treatment.

Akt, a serine/threonine kinase that functions as an oncogene, is involved in HCC cell proliferation and survival [39]. Suppression of AKT activation was implicated as a major mechanism in combinational anticancer effect of chemotherapeutic agents with several novel anticancer agents [38]–[40]. Indeed, our data showed that Smac mimetic SM-164 assisted doxorubicin and APO2L/TRAIL to repress the activation of Akt in HCC cells. Although the underlying mechanism remains to be elucidated more clearly, these data suggest that suppression of this unwanted survival signaling may contribute to the combinational anticancer activity in HCC cells.

In summary, we showed that Smac mimetics may enhance chemotherapeutic-mediated anticancer activity by enhancing apoptosis signaling and suppressing survival signaling in HCC cells. Our findings suggested that Smac mimetics offer a new approach for the therapy of chemotherapy-resistant HCC, particularly when used in combination with other drugs like APO2L/TRAIL and Doxorubicin.
